# Influence of Pregnancy in Multiple Sclerosis and Impact of Disease-Modifying Therapies

**DOI:** 10.3389/fneur.2021.697974

**Published:** 2021-07-01

**Authors:** Isabella Laura Simone, Carla Tortorella, Alma Ghirelli

**Affiliations:** ^1^Department of Basic Medical Sciences, Neurosciences and Sense Organs, University of Bari, Bari, Italy; ^2^Department of Neurosciences, San Camillo-Forlanini Hospital, Rome, Italy

**Keywords:** multiple sclerosis, pregnancy, delivery, breastfeeding, newborn, disease modifying therapy, postpartum

## Abstract

**Purpose of this Review:** This article is a systematic review on the influence pregnancy has on multiple sclerosis and the resulting impact of disease-modifying therapies.

**Findings:** Multiple sclerosis predominantly affects young women with a clinical onset most often during the child-bearing age. The impact of multiple sclerosis and disease-modifying therapies on fertility, pregnancy, fetal outcome, and breastfeeding is a pivotal topic when it comes to clinical practice. The introduction of disease-modifying therapies has changed not only the natural history of the disease but also the perspective of pregnancy in women with multiple sclerosis. Family planning requires careful consideration, especially because many disease-modifying drugs are contraindicated during pregnancy. In this article, we review current evidence collected from published literature and drug-specific pregnancy registers on the use of disease-modifying therapies. Additionally, we discuss safety profiles for each drug and correlate them to both risk for the exposed fetus and risk for the mothers interrupting treatments when seeking pregnancy.

## Introduction

Multiple sclerosis (MS) is an inflammatory disease of the central nervous system with a chronic course, mainly affecting young women, the majority of whom are of childbearing age.

Several factors have been suggested to explain the progressive increase in MS incidence in adult women in the last 30 years, including interactions between genes and environment, lifestyle modifications (contraception, diet, obesity, smoking, sunlight exposure, and vitamin D deficiency), older age at the birth of the first child, younger age at the menarche, or fewer pregnancies during a woman's lifetime (1–4).

Until the end of the 1990s, women affected by MS were frequently falsely discouraged to undertake pregnancy. Only later, the management of pregnancy in women with MS, from planning to conception and postpartum period, has been radically reviewed. The study published by Confraveux et al. (5) was pivotal in reshaping the idea of pregnancy in women with MS. Moreover, the progressive introduction of disease-modifying therapies (DMTs) has completely transformed the natural history of MS, consequently improving the perspective of pregnancy in affected women.

Several concerns afflict women who intend to plan a pregnancy, namely, the impact of the disease on fertility, the risk of transmitting MS to the progeny, the possible adverse effects of drugs on the fetus, the influence of pregnancy on MS course, the impact of the disease on the mother's ability to care for her baby, and finally, the socioeconomic burden of the disease on the family (6).

All these reasons pose an extra challenge in guiding MS women in their fertile age in making choices about pregnancy (7).

Our article is a systemic review on the influence that pregnancy has on MS and the resulting impact of DMTs. For this purpose, we performed a complete revision of literature data through MEDLINE, PubMed, and Cochrane Database, in the period from June 1982 to March 2021. MS, pregnancy, delivery, breastfeeding, newborn, disease-modifying therapy, and postpartum have been the main keywords we used to identify the most relevant studies on the topic.

## Fertility in MS

The effect MS may have on fertility is still debated. Sexual dysfunction is a common and frequent complaint in women with MS, eventually affecting their quality of life (8, 9). Reduced libido, difficulty in achieving orgasm, and dyspareunia are often reported, as well as bladder and bowel symptoms, which may affect sexual activity, interfering with social and intimate interactions in both sexes (10, 11). Furthermore, changes in sexual hormones have been reported in women with MS. High levels of follicle-stimulating hormone (FSH) and luteinizing hormone (LH) have been described, associated with low estrogen levels in the initial part of the follicular phase of the menstrual cycle, hyperprolactinemia, and hyperandrogenism (12), this latter suggesting a relationship with the slightly higher incidence of oligo/amenorrhea (13). Despite these noticeable evidences, no statistically significant association between sexual dysfunction and blood hormone abnormalities has been reported (14).

There are epidemiological studies reporting that women with MS have less children compared to the general population (15). Several factors have been suggested to interfere with parenthood, including health issues, drug-induced decreased fertility, symptoms such as fatigue, sexual or bladder dysfunction, and personal decision influenced by the disease to avoid pregnancy (16–18).

Although MS does not seem to impact on fertility, a history of infertility may be reported in women with MS, which is not necessarily linked by a cause–effect relation with the disease. In case of infertility, assisted reproductive technology (ART) becomes another relevant matter. There is evidence of an unfavorable effect of gonadotropin-releasing hormone (GnRH) agonists on disease activity, because of their effect in stimulating proliferation of leukocytes, as well as production of cytokines, chemokines, endothelial growth factor, and estrogen. On the contrary, use of GnRH antagonists would appear to be safer, although data still needs confirmation (19–22). Nevertheless, a recent study from pooled data of Boston, France, Germany, and Argentina cohorts reported an increased risk of relapses in the short-term period (3 months) after ART, both with GnRH agonists and antagonists. Furthermore, the authors suggest that continuing some MS DMTs might decrease this risk of relapse in women undergoing ART (23).

There are only few studies in literature focusing on male fertility in MS and are principally focused on sexual dysfunction, although according to one study found, males with MS present a reduced semen quality, associated with hypogonadotropic hypogonadism (24, 25).

## Impact of Pregnancy on MS Disease Activity

For what concerns the short-term impact of pregnancy on the course of the disease, several evidences point toward a positive decrease in the annualized relapse rate during pregnancy, in particular during the last trimester, which is however followed by a postpartum rebound mainly in the first 3 months after delivery (5, 26, 27). The PRIMS (Pregnancy in Multiple Sclerosis) study confirmed a decrease in annualized relapse rate (ARR) during the third trimester of pregnancy (0.2 compared to 0.7 in the year before pregnancy), as well as an increase in ARR during the first 3 months postpartum (1.2 compared to 0.7 in the year before pregnancy) so that the ARR in the post-pregnancy year was similar to that in antepartum (26). A recent study conducted in a large population-based cohort from Southern and Northern California medical centers, including 466 patients, confirmed in the entire cohort a significant lower ARR during pregnancy compared with the 2 years before conception. However, unlike previous reports, the relapse rate did not increase in the first 3 months postpartum. Moreover, the majority of women who were relapse free in the postpartum period were not taking any DMTs, and, even more surprisingly, they had only a suboptimally controlled disease at the time of conception (28).

Changes in frequency of the relapse risk both during gestation and in the postpartum period seem to be linked to fluctuations in estrogenic levels. Indeed, estrogens have a dose effect that is biphasic, since they boost the immune system at low levels, as in childbearing age, while they are immunosuppressive, hence protective, at higher levels, such as in pregnancy. The most acknowledged theory to explain the protective effect pregnancy exerts on disease activity is that, during pregnancy, estrogens with other sex hormones induce a switch in the T helper (Th) cell profile from Th1 (pro-inflammatory cytokines) to Th2 (anti-inflammatory cytokines). After delivery, the immune system gradually returns to its pre-gestation profile, which translates to disease rebound (29–33).

Early postpartum relapses notably have a poor prognostic value for what concerns MS disability progression (34). The main risk factors identified include a higher ARR in the 2 years before conception, the number of relapses during gestation, higher EDSS score at fecundation, and lastly, no history of DMT use 2 years before conception (5, 26, 35–37). Similarly, active pre-pregnancy MRI is a strong and sensitive predictor of early postpartum relapse, which is independent of clinical evidence of disease activity prior to conception and delivery (38). These clinical and MRI findings could offer neurologists a valuable strategy to minimize postpartum relapse risk in female MS patients planning pregnancy. Some studies report that women taking DMTs for a minimum of 8 weeks during pregnancy carried a decreased risk of postpartum relapses compared to patients not taking any DMT during gestation or in the trimester before conception (39). All these observations would suggest taking DMT, if safe, until conception.

There are no systematic studies on MRI activity during pregnancy, because of the risk of exposing the fetus to the thermal effect of radiofrequency as well as of intravenous contrast agent use (40, 41).

In case of relapse during pregnancy, patients should avoid corticosteroids in the first trimester, due to risk of fetal malformations such as cleft palate (42). Still, short courses of high-dose methylprednisolone are the ideal first-line treatment choice, as they are relatively safe during the second and third trimesters. However, they should be exclusively used in case of particularly severe relapses. For disabling steroid-refractory relapses, plasma exchange may be recommended, with very low associated risks (e.g., thromboembolic events).

## Impact of Pregnancy on MS Disability Progression

Another important aspect to evaluate is the impact pregnancy has on the long-term disability accumulation. Some studies showed that pregnancies have no influence on the time needed to reach a certain disability level (43–45), which instead could be predicted by a previous progressive disease course and older age at disease onset (46). On the contrary, a slower progression of disability was reported in women who conceived after disease onset, compared to the nulliparous women (47, 48). However, a bias of this latter finding could be the initial severity of the disease, which could lead the women to choose or not to become pregnant.

A recent study, including a large sample of 501 women, confirmed that pregnancy occurring after disease onset was associated with a slower disability progression only when pregnancy was analyzed as a baseline variable; conversely, this protective effect disappeared when pregnancy was considered as a time-dependent variable. The value of this study is primarily grounded on the elevated number of subjects and on the consistency of statistical analysis, which is characterized by a time-dependent approach to avoid any time-dependent bias and a propensity score to avoid selection biases (49).

Furthermore, the total number of pregnancies in an MS patient's lifetime did not appear to have a negative influence on the long-term course of the disease (26, 45, 46, 48, 50).

## Impact of MS on Pregnancy

Several studies support the evidence that MS does not impact pregnancy outcomes, which are not significantly different from the general population (51–53). A higher incidence of small for gestational age newborns, an increased predisposition to experience urinary tract infections and constipation, and more frequent interventions to induce labor, particularly in women with higher disability levels, have been reported (17, 52–55). Previous studies reported an increased rate of planned caesarean section or forceps assistance during vaginal births in MS mothers (54, 56) as well as an increased need for vacuum assistance (56, 57). On the contrary, in a large study conducted by British Columbia, mothers with MS were not more likely to receive assisted vaginal delivery or cesarean section (52).

With regard to epidural or general anesthesia, studies underline that both procedures are completely safe and they do not affect the risk of postpartum relapses (26, 58). All these observations remark that choices made during delivery have to remain with the obstetrician.

## Breastfeeding

The role of breastfeeding remains controversial. Studies on the risk of MS relapse in the period after delivery reported that breastfeeding may reduce the postpartum relapse rate (28, 36, 59–61). In this regard, Langer-Gould et al., emphasizing the protective role of exclusive breastfeeding, suggested that MS women should be encouraged to breastfeed (28). The favorable effect of breastfeeding could be mediated by immunological mechanisms related to lactational amenorrhea (62). On the other hand, studies reported that exclusive breastfeeding had no influence on postpartum relapse rate (26, 63). These controversial results for a possible protective role of exclusive breastfeeding might depend on the selection bias in some studies, such as a limited number of cases, or the lack of correction for confounding variables (e.g., number of relapses in the year before pregnancy, treatment comparison, disease duration). To our knowledge, only two of the aforementioned studies presented with the following characteristics: a large number of subjects, a time-dependent approach, and inclusion of a propensity score. Those two studies, however, reached different conclusions (60, 63). The Italian study counting 302 pregnancies (46% of which treated with DMTs, in particular interferon or glatiramer acetate) and with a postpartum follow-up period of 1 year concluded that the only factor predicting postpartum relapses was the relapse rate before and during pregnancy and not breastfeeding (63). On the other hand, the German study on 201 pregnancies (76% treated with interferons or glatiramer acetate, 11% treated with natalizumab) and with a postpartum follow-up period of 1 year concluded that exclusive breastfeeding was associated with a lower risk of postpartum relapses, while the main factor predicting disease activity after delivery was the number of relapses during pregnancy (60). The authors concluded that exclusive breastfeeding may act like a modestly effective immunosuppressant for a limited time. Differences in the number of patients undergoing treatment and in the relapse rate before pregnancy (calculated on 2 years before conception in the German study, while only on 1 year before conception in the Italian one) could account for these different conclusions. Furthermore, the benign effect of breastfeeding in reducing postpartum relapses was more evident in women with a benign disease course who chose to breastfeed compared to women with a higher disease activity who stopped breastfeeding to restart DMTs (63, 64). In this regard, it is important to underline that the necessity to restart maternal treatment with DMTs becomes an essential factor in the decision-making process for breastfeeding.

In a recent systematic review and meta-analysis of 24 studies including 2,974 women, a significant reduction of relapse rate postpartum (more than 43% reduction) was confirmed in women who were breastfeeding compared to those not breastfeeding. The studies included in this meta-analysis did not distinguish exclusive from not-exclusive breastfeeding. Therefore, conclusions about the favorable or unfavorable effect of partial breastfeeding could not be deduced (65).

All this evidence underlines how clinicians should discuss the possibility of breastfeeding with the patient, pondering both her wish and her disease activity before and during gestation (7, 66).

In the following therapy section, we argue about current recommendations for each DMT in relation to breastfeeding.

## Impact of Disease-Modifying Therapies

The introduction of DMTs in MS inevitably leads to several concerns. Clinicians and patients referring to MS centers need to discuss and balance the potential hazards of exposing the fetus to possibly teratogenic drugs vs. the maternal risk of relapses and MS progression if therapies are stopped. Fortunately, current evidence based on real-life experience in DMT use, as well as the large number of available therapies, has made easier both management and counseling of women in child-bearing age. The mechanism of action and adverse effect profiles of each DMT are classified and continuous reviewed by the Food and Drug Administration (FDA) and European Medicine Agency (EMA), which evaluate drugs according to their risk weighted against potential benefit.

The impact of DMTs on fertility is summarized in Table 1.

**Table 1:** Impact on fertility induced by disease-modifying therapies in multiple sclerosis.

**Drug**	**Effect on fertility**
Interferon beta	No effect on fertility.
Glatiramer acetate	No effect on fertility.
Dimethyl fumarate	In animal studies reduction of estrogen, but no effect on fertility.
Fingolimod	No effect on fertility.
Siponimod	No effect on fertility.
Teriflunomide	In male animal studies reduction of sperm count, but no effect on fertility.
Cladribine	In male animal studies reduction of germ cells and sperm count, but no effect on fertility.
Natalizumab	In animal studies reduction in fertility. No data in humans.
Alemtuzumab	In animal studies reduction in corpora lutea and implantation in uterus. No data in humans.
Ocrelizumab	No effect on fertility.

Population-based studies showed that among MS patients who became pregnant, more than 40% were not taking DMTs in the 12 months before conception, suggesting that many women prefer to avoid any risk of drug-induced adverse outcomes for their fetus. Furthermore, women with little or no disability, rare relapses, and low lesion burden load on MRI or who required low effective therapies to control disease activity in the past were the most likely to interrupt treatment during and after pregnancy (28).

Neurologists should discuss with their patients about the benefit/risk profile of DMTs before, during, and after pregnancy at or soon after MS diagnosis, and then discussions should be regularly repeated afterward. The choice of optimal time for a woman with MS to become pregnant should be evaluated individually, according to her disease activity, her response to drugs, and the availability of resources to manage the motherhood. As such, family planning should be a crucial step for women of reproductive age with MS, and they should undergo regular counseling on the use of effective contraception in order to plan pregnancies.

Reliable contraception is recommended for patients taking DMT, but it is tailored on each drug. A systematic review was performed to estimate the safety of contraceptive use in MS patients (67). The four studies selected by the authors of the review concluded that the use of combined oral contraceptives (type not specified) did not worsen the clinical–neuroradiological course of the disease (defined by disability level, disease severity or progression, relapse, or number of new brain lesions on MRI after 96 weeks of follow-up) (67–71). The US Medical Eligibility Criteria for contraceptive use in MS women reported that most contraceptive methods are safe—the only exception being use of contraceptives in MS patients with prolonged immobility due to concerns on venous thromboembolism risk (72).

### Self-Injectable DMTS

EMA and FDA recommendations for the management of self-injectable DMTs in pregnancy and breastfeeding are summarized in Table 2.

**Table 2:** EMA and FDA recommendations for the management of self-injectable DTMs in pregnancy and breastfeeding.

**Treatment**	**EMA**	**FDA**	**Milk secretion**	**Clinical practice**
Interferons beta	**Pregnancy**: initiation of treatment contraindicated during pregnancy. Update of EMA in 2019 allows to consider continuing IFNβ-1a until conception and during pregnancy as clinically needed. **Breastfeeding**: no harmful effects on breastfed infants are anticipated; can be used during breastfeeding.	**Pregnancy**: Should be used during pregnancy only if the potential benefit justifies the potential risk to the fetus. **Breastfeeding:** administer with caution to a nursing mother.	Not known.	Continue until pregnancy confirmed. In selected patients with highly active disease, may be administered throughout pregnancy after careful evaluation of the risk–benefit ratio.
Glatiramer acetate	**Pregnancy**: pregnancy contraindication removed from the EU label in 2017. **Breastfeeding:** decide on the balance between infant breastfeeding versus maternal therapy.	**Pregnancy:** Only use during pregnancy if clearly needed. **Breastfeeding:** consider benefits of breastfeeding against possible risks to the fetus.	Not known.	Continue until pregnancy confirmed. Continued use in pregnancy now supported in some cases.

*EMA, European Medicines Agency; FDA, U.S. Food and Drug Administration*.

### Interferons Beta and Glatiramer Acetate

Interferons β (IFNβs) were the first DMTs to be approved in MS. Their mechanism of action is pleiotropic. They induce the shift in T cell balance toward the anti-inflammatory profile of Th-2 cells, as well as inhibition of T-cell migration blocking metalloproteases and adhesion molecules (73).

Glatiramer acetate (GA) is a mixture of four synthetic polypeptides (l-glutamic acid, l-lysine, l-alanine, and l-tyrosine), like the myelin basic protein. Although its precise mechanism of action is still unknown, GA has been reported to induce a shift from Th1 to Th2 responses, with an increase in T-regulatory cells and downregulation of both Th1 and Th17 cells (73).

#### Fertility

Studies on the impact of IFNβs and GA on the fertility are rare. Clinical trials on IFNβ-1b report a similar rate of pregnancies in both treatment and placebo groups (74, 75). In addition, IFNβ or GA showed no alterations on sperm count (76, 77).

#### Pregnancy and Fetal Development

Several evidences suggest that IFNβs and GA do not increase the risk for spontaneous abortions, preterm birth, or major congenital malformations. Indeed, registry-based and post-marketing studies on a large series of pregnant women exposed to IFNβ do not report any increased risk of either spontaneous abortions or congenital malformations in newborns compared to the general population (78–82).

Some studies reported a lower body weight in newborns (39, 78, 83, 84), which was not confirmed by others (85, 86).

European prescribing information has been updated in 2019; it now allows considering continuing IFNβ-1a at dose of 44 mcg (Merck-Serono®) until conception, during pregnancy as clinically needed, and while breastfeeding^1^.

For what concerns GA, animal studies did not report evidence of teratogenicity, fetal development, or malformations. One study on a small number of patients reports a reduced birth length of 2.3 cm in newborns exposed to GA during the first trimester of pregnancy (36). On the contrary, post-marketing surveillance in a large sample of pregnancies confirms the safety of the treatment, also when exposure occurred in the first trimester (86–90).

Recent data collected by Teva Pharmaceuticals as part of a global pharmacovigilance database provided important evidence on the safety of branded GA during gestation, highlighting the lack of teratogenic effects (91). For these reasons, GA is not contraindicated during pregnancy, if the maternal benefit outweighs the risk to the fetus. Reports on GA exposure during the entire gestation are rare; however, no increased risk of an adverse pregnancy outcome has been disclosed (88, 92, 93). EMA has withdrawn pregnancy contraindication to Copaxone 40 mg/ml (Teva Pharmaceuticals®) in 2017.

No relation has been documented between paternal exposure to IFN beta or GA at the time of fecundation and the risk of adverse outcomes (76, 77).

#### Breastfeeding

The transfer of IFNβs and GA into breast milk is very unlikely because of their large molecular weight and high polarity (94). Use of IFNβ-1a (Rebif®) is indeed approved by EMA during breastfeeding ^1^.

Based on these evidences, the discontinuation of IFNβ or GA during pregnancy may be avoided in MS patients with a high level of disability. On the other hand, treatment continuation might lead to a reduced risk of relapses postpartum, even if there is no data about this.

### Oral Drugs

EMA and FDA recommendations for management of oral DMTs in pregnancy and breastfeeding are summarized in Table 3.

**Table 3:** EMA and FDA recommendations for the management of oral drugs in pregnancy and breastfeeding.

**Treatment**	**EMA**	**FDA**	**Milk secretion**	**Clinical practice**
Dimethyl fumarate	**Pregnancy:** not recommended during pregnancy and in fertile women not using appropriate contraception. Should be used only if clearly needed and if the potential benefit justifies the potential risk to the fetus. **Breastfeeding**: decide on the balance between infant breastfeeding versus maternal therapy.	**Pregnancy**: Use only if the potential benefit justifies the potential risk to the fetus. **Breastfeeding**: consider benefits of breastfeeding against possible risks to the fetus.	Not known.	Discontinue before conception and maintain effective contraception for an appropriate time before pregnancy. Monitor disease activity with MRI, cease breastfeeding if applicable and resume therapy.
Fingolimod	**Pregnancy:** women should not become pregnant and active contraception is recommended. Since it takes approximately 2 months to eliminate fingolimod from the body, contraception should be continued for 2 months after drug cessation before looking for pregnancy. **Breastfeeding:** contraindicated.	**Pregnancy:** Use effective contraception during treatment and for 2 months after interruption. Use only if the potential benefit justifies the potential risk to the fetus. **Breastfeeding:** consider benefits of breastfeeding against possible risks to the fetus.	Yes, in animals.	Discontinue before conception and maintain effective contraception for an appropriate period of time. Monitor disease activity with MRI, cease breastfeeding if applicable and resume therapy.
Siponimod	**Pregnancy**: contraindicated during pregnancy. Fertile women must have a negative pregnancy test, and they should use effective contraception during treatment and for at least 10 days after discontinuation. ***Breastfeeding***: contraindicated.	**Pregnancy**: contraindicated; women should not become pregnant for at least 10 days after drug cessation. **Breastfeeding**: consider benefits of breastfeeding against possible risks to the fetus.	Yes, in animals.	Discontinue therapy at least 10 days before conception while maintaining effective contraception. Breastfeeding is contraindicated.
Teriflunomide	**Pregnancy:** contraindicated. Use accelerated drug elimination procedure if planning pregnancy or pregnancy occurs on treatment. **Breastfeeding:** contraindicated.	**Pregnancy:** contraindicated: use accelerated drug elimination procedure if planning pregnancy or pregnancy occurs on treatment. **Breastfeeding:** women should not breastfeed while on treatment.	Yes, in animals.	Use effective contraception during treatment and after treatment as long as drug plasma concentration is above 0.02 mg/l. Breastfeeding is contraindicated.
Cladribine	**Pregnancy:** contraindicated. Women should not become pregnant for at least 6 months after the last dose. **Breastfeeding:** contraindicated. Women should not breastfeed for at least 1 week after the last dose.	**Pregnancy:** contraindicated. Women should not become pregnant for at least 6 months after the last dose. **Breastfeeding:** contraindicated. Women should not breastfeed for at least 10 days after the last dose.	Not known.	Women should not become pregnant for at least 6 months after the last dose. Women who become pregnant under therapy should discontinue treatment. Breastfeeding is contraindicated.

*EMA, European Medicines Agency; FDA, U.S. Food and Drug Administration*.

### Dimethyl Fumarate

Dimethyl fumarate (DMF) is an oral drug approved for the treatment of relapsing–remitting MS (RRMS). DMF decreases the absolute lymphocyte count, mainly affecting CD8^+^ T cells but also CD4^+^ T cells, B lymphocytes, myeloid, and natural killer populations, which all shifted toward an anti-inflammatory state. Furthermore, *in vitro* and animal models demonstrated that DMF promotes neuronal survival within the central nervous system (CNS) by acting on an Nrf2 pathway, with consequent antioxidative, anti-inflammatory, and cytoprotective effects (95).

#### Fertility

In female rats, high doses of DMF may induce a reduction in estrogen levels, however not affecting fertility (96). In male rats, no evidence of impaired fertility was reported (97).

#### Pregnancy and Fetal Development

The drug is able to cross the placental barrier. In animal studies, low birth weight, delayed ossification, and a higher risk for spontaneous abortion at very high and toxic doses were registered (97).

Human data are too sparse to draw conclusions. An international registry is currently tracing gestations in women exposed to DMF. A rate of premature fetal death of 9% emerged in a recent report from this database (194 pregnancies with known outcome), with a rate of birth defects of 4% (98). Therefore, women of childbearing age are recommended to use contraception while being treated with DMF and switching to an alternative DMT should be contemplated depending on the degree of disease activity. In general, it is recommended to stop DMF with the plan to conceive, and DMF received a pregnancy category 2 by EMA (96)^2^. However, due to its very short half-life (1 h) and its almost negligible tissue accumulation, DMF is quickly eliminated, and no washout period is required after drug discontinuation when seeking pregnancy, even though other studies suggest establishing a washout period of 2 weeks (99). DMF should be immediately stopped after discovery of unexpected pregnancy during treatment, and fetal organ screening ultrasound might be considered (100). Lastly, drug agencies have not provided any recommendation regarding paternal exposure to DMF at the time of conception and the consequent risk of adverse outcomes (101).

#### Breastfeeding

To our knowledge, there are currently no data regarding excretion of DMF or its metabolite in breast milk. Only low amounts of the active metabolite of DMF were found in breast milk, and therefore, no adverse effects in breastfed infants should be expected. However, some authors as well as FDA and EMA recommend avoiding breastfeeding while on therapy (96, 102)^2^. The benefit of breastfeeding for the child and the benefit of receiving therapy for the woman should be taken into account in individual cases.

### Fingolimod

Fingolimod (FTY720; Gilenya®) is the first oral drug approved for RRMS treatment. Fingolimod is a sphingosine-1-phosphate (S1P) receptor modulator regulating lymphocyte egression from lymphoid tissues into the circulation. Furthermore, S1P1, S1P2, and S1P3 receptors, being expressed in the endothelial and vascular smooth muscle cells during embryonic development, may regulate vascular development. Finally, the S1P1 signaling pathway is fundamental in neurogenesis and the subsequent development of the nervous system (103, 104).

#### Fertility

In animal studies, fingolimod demonstrated no effect on fertility of both male and female rats, even at doses approximately 200 times higher than the recommended dose in humans (105, 106).

#### Pregnancy and Fetal Development

Fingolimod can cross the placental barrier, and it was found causative of teratogenic effect in rats, such as persistent truncus arteriosus and ventricular septal defect. In humans, it likely increases the risk of malformations. According to regulatory agency recommendations, fingolimod is contraindicated during pregnancy. Recommended washout is 2 months before pregnancy (105, 106).

The clinical development fingolimod program included 89 exposed pregnancies. Pregnancy was considered “exposed to the drug” if fingolimod was ongoing at conception or 6 weeks before. Spontaneous abortion occurred in 24% of pregnancies, slightly exceeding the rate registered in the general population. Abnormal fetal development was reported in 7.6% of cases, being borderline considering normal values expected in the general population (4–8%). Fetal development abnormalities included one case of acrania, one case of unilateral congenital postero-medial bowing of the tibia, and one case of tetralogy of Fallot, and they were all associated with fetal exposure to fingolimod in the first 3 months of pregnancy (107). More recently, an additional 717 pregnancies exposed to fingolimod were collected. In this cohort, the prevalence of major cardiac abnormalities was comparable with that in the general population. The overall percentage of spontaneous (15%) and elective abortion was within the expected range (108).

Contraception is recommended during treatment with fingolimod and in the 2 months after discontinuation (106). In case of accidental exposure to fingolimod after suspension, organ screening ultrasound should be recommended.

In clinical practice, bridging with a depleting agent or natalizumab should be considered. When fingolimod is withdrawn before pregnancy, the risk of disease activity rebound must be taken into account, even if the magnitude of this risk is not known yet, as well as the successful strategies to minimize the risk (109, 110). Natalizumab might be considered for bridging strategies, and an extended dosing regimen is usually proposed in order to guarantee a lower exposure of the fetus to the drug and a lower PML risk for the mother. Natalizumab should be stopped anyway at least at 34 weeks of pregnancy; it is eventually administered in an off-label setting.

#### Breastfeeding

In animal studies, fingolimod was found to be excreted in rat milk at concentrations 2–3-fold higher than in maternal plasma (101). Excretion in human breast milk is still unknown, but probable (106). For this reason, fingolimod is not compatible with lactation.

### Siponimod

Siponimod is a new S1P modulator targeting S1P1 and S1P5 receptors. The molecule is characterized by a molecular weight of 516 DA and a half-life of approximately 30 h, and it is contraindicated in carriers of the CYP2C9_3/_3 genotype (111). FDA has approved siponimod for the treatment of adult patients with RRMS, active secondary progressive MS, and clinically isolated syndrome, whereas it has been indicated by EMA for the treatment of secondary progressive MS with clinical or MRI active disease (112)^3^.

#### Fertility

Animal studies failed to demonstrate any noxious effect on male reproductive organs in rats and monkeys. No alterations in fertility on female rats were demonstrated either (112)^3^.

#### Pregnancy and Fetal Development

Placental passage of siponimod and its metabolites has been demonstrated in animal studies. Siponimod induced embryotoxicity and fetotoxicity in rats and rabbits as well as teratogenicity in rats. Embryonic and fetal deaths, along with skeletal or visceral defects (e.g., urogenital) at exposure levels similar to human dosages (daily dose of 2 mg), have been reported in rats and rabbits (112)^3^.

There are a limited number of studies available on the use of siponimod in pregnant patients. Since fetal threat has been demonstrated by animal studies, siponimod is contraindicated during gestation and in fertile women not using effective contraception. Before initiating treatment, fertile women must be educated on the serious fetal risks associated with the drug, and it is recommended to use effective contraceptive measures both during treatment and for at least 10 days after drug suspension^3^. If pregnancy occurs while on treatment, siponimod must be immediately discontinued and medical advice on the risk of possible fetotoxicity should be given. Furthermore, ultrasound investigations should be performed.

When stopping siponimod in order to plan a pregnancy, the risk of disease activity rebound should be carefully considered.

#### Breastfeeding

No published data is currently available on the excretion of siponimod in human milk, on the effects of the drug on the breastfed infant, or on milk production itself. A study in lactating rats has demonstrated that siponimod and/or its metabolites are excreted in milk; hence, siponimod has been contraindicated during breastfeeding (112)^3^.

### Teriflunomide

Teriflunomide is an oral immunomodulatory drug taken once daily and approved for RRMS. The drug interferes with *de novo* pyrimidine synthesis by specific inhibition of the mitochondrial enzyme dihydro-orotate dehydrogenase (DHODH), which is highly expressed in proliferating lymphocytes (113).

#### Fertility

Studies in animal models have failed to show adverse effects on male or female fertility. Although a small reduction in sperm count has been reported in rats at highest teriflunomide doses, no effects on fertility have been demonstrated (114, 115).

#### Pregnancy and Fetal Development

Drug-induced embryotoxicity and teratogenicity, with the occurrence of abnormalities of the axial skeleton and the head (e.g., microphthalmia, hydrocephaly), have been reported in animal studies (114, 115).

For this reason, although human data on teratogenicity are lacking, teriflunomide is contraindicated by FDA in the 3 $\frac{1}{2}$ months before pregnancy and during pregnancy (115).

A recent review on the outcomes of 222 pregnancies exposed to teriflunomide in the first trimester based on clinical trials and post-marketing experiences reported a frequency of major birth defects consistent with that in the general and MS population. Similarly, the incidence of spontaneous abortions (21.2% overall) was similar to that reported in the general population (15–20%) (116).

For what concerns male MS patients treated with teriflunomide, pregnancies of their female partners resulted in 12.5% of spontaneous abortions, 4.2% elective abortions, and two cases of fetal anomaly (116).

Teriflunomide is a small molecule with a MW of 270 g/mol, which is rapidly and completely absorbed after oral administration. Consistent with the extensive plasma protein binding of teriflunomide, its elimination half-life is approximately 10–12 days, but to reach total elimination of the drug, 8–24 months may be required (113). Therefore, when planning a pregnancy, a washout strategy may be proposed with either cholestyramine (8 g for three times daily or, if 8 g is not supported, 4 g for three times daily, for 11 days) or activated charcoal (50 g, two times daily for 11 days) in order to accelerate the elimination of the treatment. Fertile women taking teriflunomide must use effective contraception during and after treatment as long as the drug plasmatic concentration is above 0.02 mg/l (115).

Teriflunomide can be identified at low concentrations in semen (117). According to FDA, men wishing to father a child should suspend treatment and undergo accelerated washout (115). On the contrary, EMA states that the risk of male-mediated embryonic or fetal toxicity induced by teriflunomide is negligible (114).

#### Breastfeeding

Being a small molecule, teriflunomide is likely excreted into breast milk. Therefore, its administration is contraindicated during lactation (114, 115).

### Cladribine

Cladribine (2-chlorodeoxyadenosine, 2-CdA) is a synthetic chlorinated analog of deoxyadenosine that interferes with DNA synthesis. It induces a prolonged lymphocyte depletion, which is more selective toward B lymphocytes (118).

The drug is a small molecule with a MW of 285, and it represents an example of oral selective pulse immune reconstitution therapy. Cladribine received approval by FDA for RRMS and active secondary progressive MS and by EMA for highly active relapsing MS (119, 120).

#### Fertility

Different reproductive toxicology studies carried out in animals (unpublished results, Merck KGaA) failed to demonstrate a role for cladribine in impacting female and male fertility, or in affecting peri–postnatal development abnormalities in the offspring. However, cladribine induced depletion of germ cells, spermatids, and spermatozoa in mice. Studies of ovarian dysfunction induced by cladribine are lacking; it can even induce DNA strand breaks (119, 120).

#### Pregnancy and Fetal Development

Cladribine showed teratogenicity in mice and rabbits when given intravenously. In humans, the half-life of the drug is short (<24 h), with a rapid elimination after administration (121). However, considering that animal studies demonstrated both teratogenicity at doses higher than those administered to humans, as well as short-term effects on male germ cells, caution should be exerted during and after cladribine dosing.

Data from clinical programs on outcomes from women exposed to cladribine (*n* = 44) and from women whose partners had been exposed to cladribine are very limited. Eighteen of 44 pregnancies exposed to cladribine were carried to term; nine were terminated by spontaneous abortions, three by induced abortions mainly because of ectopic pregnancy and choriocarcinoma. The female partners of nine male patients treated with cladribine had a total of 10 pregnancies, nine of which were carried to term with resulting live births. No congenital malformations were reported (122).

There are no published data from post-marketing clinical studies on the effect of cladribine tablets in pregnancy.

Cladribine is contraindicated in pregnancy (119, 120). The drug crosses the placental barrier. Based on its potential for serious fetal risk, manufacturers recommend adding a barrier method of contraception (even if already on hormonal contraception) during cladribine treatment and for at least 4 weeks after the last dose, to be repeated at every course of treatment. According to current European labeling recommendations, women should not become pregnant for at least 6 months after a course of cladribine; similarly, male patients must adopt effective contraception to prevent pregnancy of their partner during cladribine treatment and for at least 6 months after the last dose (119).

#### Breastfeeding

Whether cladribine is excreted in human milk is unknown. However, given the potential for serious adverse effects, women should not breastfeed during treatment and for at least a week after the last drug administration (119, 120).

### Injectable Monoclonal Antibodies

EMA and FDA recommendations for the management of injectable monoclonal antibodies in pregnancy and breastfeeding are summarized in Table 4.

**Table 4:** EMA and FDA recommendations for the management of injectable monoclonal antibodies in pregnancy and breastfeeding.

**Treatment**	**EMA**	**FDA**	**Milk secretion**	**Clinical practice**
Natalizumab	**Pregnancy:** if a woman becomes pregnant, discontinuation of the drug should be considered. Newborns of women exposed to natalizumab during the third trimester of pregnancy should be monitored for potential hematologic abnormalities. **Breastfeeding:** contraindicated. Breastfeeding should be discontinued during treatment.	**Pregnancy:** use during pregnancy only if the potential benefit justifies the potential risk to the fetus. **Breastfeeding:** consider benefits of breastfeeding against possible risks to the fetus.	Yes, in humans.	Continue until pregnancy confirmed. Continue until second trimester of gestation in highly active disease. Resume therapy as soon as possible after delivery.
Alemtuzumab	**Pregnancy:** maintain contraception for 4 months after the last dose. Only use during pregnancy if potential benefit justifies potential risk to the fetus. **Breastfeeding:** avoid breastfeeding during and for 4 months after each treatment course (but balance potential benefit of breastfeeding with potential risks from exposure).	**Pregnancy:** use during pregnancy only if the potential benefit justifies the potential risk to the fetus. Maintain contraception for at least 4 months after last dose. **Breastfeeding:** consider benefits of breastfeeding against possible risks to the fetus.	Yes, in animals.	Discontinue before conception and maintain effective contraception for an appropriate period of time before pregnancy.
Ocrelizumab	**Pregnancy:** fertile women should use contraception while receiving ocrelizumab and for 12 months after the last infusion. **Breastfeeding:** advise women to discontinue breastfeeding during treatment.	**Pregnancy:** there are no adequate data on the developmental risk associated with use of ocrelizumab in pregnant women. Fertile women should use contraception while receiving the drug and for 6 months after the last infusion. **Breastfeeding:** consider benefits of breastfeeding against possible risks to the fetus.	Yes, in animals.	Discontinue before conception and maintain effective contraception for an appropriate period before pregnancy. Monitor disease activity with MRI, cease breastfeeding if applicable, and resume therapy

*EMA, European Medicines Agency; FDA, U.S. Food and Drug Administration*.

### Natalizumab

Natalizumab (NTZ) is a monoclonal antibody approved for the treatment of highly active RRMS. It reduces CNS inflammation by blocking very-late antigen (VLA)-4 on the surface of lymphocytes, thus preventing their transmigration through the blood–brain barrier (BBB) (123).

#### Fertility

Preclinical studies demonstrated a reduction in female guinea pig fertility at dose levels (30 mg/kg) of NTZ 2.3 times higher than the clinical dose (124). Male guinea pig fertility was unaltered. No studies have analyzed the effects of natalizumab on human fertility (125, 126).

#### Pregnancy and Fetal Development

NTZ is recognized to have established teratogenic effects in animal models. In humans, adequate and well-controlled studies are lacking.

Being a very large molecule with a MW 150 kDa, NTZ is unable to cross the placenta during the first trimester of pregnancy (123), but it can reach fetal circulation, being carried by active transportation through the placenta, from the second trimester.

In animal studies on guinea pigs and cynomolgus monkeys, NTZ did not show fetotoxicity or teratogenicity (127, 128). However, preclinical studies demonstrated that an exposure to NTZ throughout pregnancy can cause hematological effects in the offspring, which were anyway reversible after drug elimination (128).

In humans, conflicting results for increased abortion rates have been reported, but no specific patterns of malformations suggesting a drug effect emerged (129–131). Effects on spontaneous abortion were also confirmed in an Italian population of 92 pregnancies exposed to the drug, demonstrating that NTZ exposure to up to 12 weeks of gestation was associated with spontaneous abortion (odds ratio [OR] 3.9, 95% confidence interval [CI] 1.9–8.5, *p* <0.001) if compared to IFNβ exposure or no exposure. The rate of spontaneous abortion (17.4%) was anyway within the limits expected in the general population (132).

On this background, avoiding pregnancy during treatment and a post-treatment washout period of at least 3 months before conception is recommended by regulatory agencies^4^. Nevertheless, maternal risk of disease reactivation might be considered and weighted in respect to fetal risk. A disease reactivation or true disease rebound is described in one-third of patients within 2–6 months after NTZ suspension (133–135), and the gestational period seems protective against disease reactivation (136, 137). When approaching the use of NTZ during pregnancy, clinicians always need to consider that MS patients treated with NTZ usually have aggressive MS requiring a very active drug. A recent meta-analysis tried to identify patients that were at a higher risk of post-NTZ suspension disease reactivation. It resulted that younger age, higher number of relapses, gadolinium-enhanced lesions before treatment initiation, and fewer NTZ infusions were associated with increased risk of disease reactivation after NTZ (*p* ≤ 0.05) (137). For this reason, an individualized evaluation of the risk-to-benefit ratio must be considered for each patient treated with NTZ when planning pregnancy.

Recent studies suggest that NTZ should be considered as a therapeutic option in pregnant patients with highly active MS. Haghikia et al. report a case series of 13 pregnancies in 12 women with highly active MS who were treated with NTZ in the last trimester of pregnancy. Mild to moderate hematologic alterations were observed in 10 of 13 infants, such as thrombocytopenia and anemia. In the majority of the newborns, these hematological aberrations resolved during the 4 months after birth and no specific treatment were needed, although a subclinical bleeding complication was reported. In a subsample of five mother–child pairs, natalizumab was detected in the umbilical cord blood of the newborns. Pediatricians, at delivery, should be prompt to evaluate potential signs of anemia and thrombocytopenia in newborns exposed to natalizumab during the third trimester of pregnancy (138). Triplett et al. suggested that in order to reduce possible hematological complications of the newborn, NTZ doses could be modified during the third trimester, while prenatal umbilical cord should be sampled, and intravenous immunoglobulins should be administered (139).

Since NTZ suspension is associated with a high risk of disease reactivation, pregnancy could be planned without interrupting the drug and with a strict monitoring of conception.

In practice, a patient-tailored approach is suggested which can be either:

1. *Conservative approach*: discontinue natalizumab prior to conception and maintain contraception for 2–3 additional months after discontinuation;
2. *Semi-active approach:* maintaining natalizumab at least until conception (test beta-HCG before each infusion, 6–8 weeks extended dosing regimen) and restarting treatment early after delivery;
3. *Active approach*: maintaining natalizumab until the 30–34th weeks of pregnancy (6–8-week extended dosing regimen) and early restarting after delivery (8–12 weeks after last infusion);
4. *Bridging approach*: shifting natalizumab to a depleting agent (rituximab or ocrelizumab).

Current consensus UK guidelines recommend an active approach (140). Even if evidence supporting the aforementioned approaches is available, the use of NTZ, as well as depleting agents, during pregnancy remains off label in clinical practice. Therefore, the adoption of any approach must always be shared with the patient and a report of the discussion annotated in clinical records.

#### Breastfeeding

NTZ is excreted into human breast milk. Although NTZ is not orally bioavailable, the effects of exposure to infants are unknown. One study reported that the transfer of natalizumab into human milk increased over time and with repeated injections, with the highest concentration of 2.83 μg/ml at day 50 and with a relative infant dose of 5.3% (141). For this reason, NTZ should be avoided during lactation, also according to EMA and FDA prescribing information (125, 126, 142). However, the risk/benefit ratio of breastfeeding in case of restarting NTZ after delivery must be discussed, considering that reliable data are lacking (143). If women decide to breastfeed under natalizumab, infants should be monitored for hematological abnormalities.

### Alemtuzumab

Alemtuzumab (ALZ) is an anti-CD52 humanized monoclonal antibody that is administered annually, typically used in aggressive or refractory MS. The drug provokes depletion of B and T lymphocytes (144, 145), in regulatory T- and B cells, as well as in secreting cytokines with a less inflammatory profile (146, 147).

#### Fertility

Animal production studies showed an adverse effect on fertility. In female mice, intravenous infusion of ALZ at doses up to 10 mg/kg/day (4.7 times above the daily dose recommended in humans) for five consecutive days produced a significant reduction of corpora lutea and implantation sites per female mouse. No effects on fertility in male mice were reported. Adequate clinical safety data on fertility in humans (women and men) are lacking (148, 149).

#### Pregnancy and Fetal Development

Animal studies reported an increased embryonic lethality and a reduced level of B and T circulating lymphocytes in the offspring when pregnant mice were exposed to ALZ during the period of organogenesis (148, 149). Data from the clinical development programs on 264 pregnancies, occurring in 160 out of 972 women treated with 12 or 24 mg ALZ before conception, showed normal live births, without increase in congenital anomalies or birth defects. Furthermore, the incidence of spontaneous abortion was not different from that reported in the general population and in treatment-naive MS patients (150). For what concerns the risk of postpartum relapse, treatment with ALZ induced a significant reduction in the ARR postpartum (0.2) as compared to the rate before treatment (1.7) or that reported in literature, which ranges from 2.0 to 0.5 (5, 35, 151). This data confirms the prolonged clinical effect of ALZ on the risk of disease activity. To our knowledge, there are no published post-marketing studies to date concerning ALZ exposure during pregnancy, except for two ECTRIMS abstracts. On 18 pregnancies exposed to ALZ (last ALZ <4 months before last menstrual period), 7.7% pregnancies ended in spontaneous abortion, 8.3% babies were affected by malformations or genetic abnormalities, and 8.3% were born preterm. A favorable disease course without relapses during pregnancy and postpartum was reported in all women with term pregnancies (152).

According to EMA and FDA prescribing information, ALZ is contraindicated in pregnancy (148, 149).

Monoclonal antibodies, such as ALZ, are known to cross the blood–placenta barrier, at least certainly after 20 weeks of gestation. Therefore, their use during pregnancy may potentially affect the fetus. ALZ concentration becomes low or undetectable in plasma approximately 30 days after a course of treatment (153). Despite this, recommendations from manufacturers are to avoid conception and to use effective contraception for 4 months following a course of treatment with ALZ. According to current European and USA labeling recommendations, ALZ should be administered during gestation only if potential maternal benefits justify the possible risk to the fetus. Women have to be informed on the possible drug-induced risks, which include autoimmune thyroid disease (37%), immune thrombocytopenic purpura (1%), Goodpasture syndrome (0.1%), and other autoimmune diseases that may persist for 4 years after a cycle of ALZ. If those autoimmune disorders occur during gestation, they may distress both the mother and the fetus as antibodies cross the placenta (e.g., neonatal thyrotoxicosis) (139, 154). In case of maternal autoimmune thyroid diseases, thyroid hormones have to be monitored monthly during pregnancy. These drug-induced autoimmune diseases, and in particular consequent hypothyroidism, might increase the risk of spontaneous abortions, intrauterine growth retardation, preeclampsia and preterm birth, irregular menstruation, infertility, and delayed mental development of the child (149).

#### Breastfeeding

Alemtuzumab, being a monoclonal antibody, can be excreted in human milk. Therefore, breastfeeding is discouraged for at least 4 months after the last infusion of the drug in each treatment course.

### Ocrelizumab

OCR is a humanized anti-CD20 monoclonal antibody that depletes B cells through antibody-mediated and complement-mediated cellular cytotoxicity. The B-cell depletion is evident within 14 days of infusion, while the B-cell population recovers in 72 weeks (155). The drug is approved by EMA and FDA for treating adult patients with RRMS or early primary progressive MS (PPMS) with MRI findings of inflammatory activity (156, 157).

#### Fertility

Data from animal studies showed no effects on reproductive organs in male monkeys as well as on estrus cycle in female monkeys that were administered by intravenous OCR at 2 and 10 times the recommended human dose of 600 mg (157).

#### Pregnancy and Fetal Development

Animal studies reported both teratogenicity and fetotoxicity. In two pre- and postnatal development studies carried out in cynomolgus monkeys, the administration of OCR throughout gestation was correlated with glomerulopathy, lymphoid follicle formation in bone marrow, lymphoplasmacytic renal inflammation, and decreased testicular weight in the offspring. There are five cases of neonatal moribundities caused by opportunistic bacterial infection impacted by B-cell depletion. Animal offspring born from mothers exposed to OCR exhibited depleted B cell populations after delivery. No teratogenic effects were reported in animal studies (156).

Data on the safety profile of OCR before and during pregnancy in women are limited. Preliminary data on pregnancy outcomes were reported from OCR clinical trials and post-marketing sources. Out of 267 pregnancies exposed to OCR (dose range 20–2,000 mg), 62 live births, 86 ongoing pregnancies, 25 elective abortions, 10 spontaneous abortions, 1 stillbirth, 3 ectopic pregnancies, 22 lost to follow-up, and 58 unknown outcomes have been reported. The outcomes of these cases do not suggest an increased risk of adverse pregnancy outcomes. No data on B cell count have been published in newborns and infants exposed to OCR during gestation (158).

A recent cohort study on treatment with anti-CD20 (OCR or rituximab) in women with MS or neuromyelitis optica spectrum disorder reported that pregnancy outcomes after treatments have been administered in the years before pregnancy were similar to those expected in the general population. On the contrary, treatment given during pregnancy could result in more preterm births and congenital malformations.

For what concerns disease activity, anti-CD20 treatment induced a significant decrease in the number of relapses during pregnancy and in the postpartum period (159). Considering that OCR may cross the placental barrier, the manufacturers recommend that fertile women should use adequate contraception while receiving the drug. In Europe, current recommendations suggest planning pregnancy only after 12 months after the last infusion of OCR (compared to the 6-month interval recommended by FDA). OCR should be avoided during pregnancy unless “the potential benefit to the mother outweighs the potential risk to the fetus” (156, 157).

#### Breastfeeding

Published data in animals have demonstrated excretion of OCR in breast milk, with measurable levels of OCR (approximated 0.2% of serum levels) during the lactation period. Reliable data on human are lacking. Recently, a study with another anti-CD20 (i.e., rituximab) reported that levels of the drug in milk were <240 times the amount detected in maternal serum, suggesting that this minimal excretion was related with the drug's pharmacological property, with monoclonal antibodies being macromolecules, and therefore the breastfeeding would be allowed (160). However, EMA and FDA recommend that women should be advised not to breastfeed during or 6 months after discontinuing the treatment (156, 157).

## Conclusions

This review attempts to summarize current evidence and expert recommendations about specific issues regarding pregnancy planning, pregnancy course, partum and postpartum period, breastfeeding, and the management of DMT use in MS women. Based on current evidence, MS does not impact the fertility in either sex, or the women's ability to conceive and to carry the fetus to term. The disease does not increase the risk of spontaneous abortion, malformations, and caesarean delivery. Pregnancy appears to be protective against MS disease activity, particularly during the third trimester, but an increased risk of relapse is reported in the first 3 months postpartum. Pregnancies do not impact either the long-term disease course or the accumulation of disability. Results from registers, real-world databases, and pharmacovigilance have increased our awareness on the impact DMTs exert on the pregnancy. Consequently, family planning strategies for patients with MS have changed. Women with MS should be supported and encouraged to have children and to breastfeed, also considering the possible favorable effect of exclusive breastfeeding. Neurologists and patients should tailor together the best therapy for any pregnant woman, considering the chances of conception in relation to DMTs without exposing the fetus to any possible risk and the safety of a benign postpartum period. Specific recommendations regarding whether and when to discontinue DMTs or switch to other therapy are continuously evolving, which is why neurologists are required to be constantly updated with both literature and international guidelines.

## Author Contributions

IS: paper design and conception. IS, CT, and AG: manuscript writing. AG: figures. IS: manuscript revision and editing. All the authors agreed to be accountable for the content of the work.

## Conflict of Interest

The authors declare that the research was conducted in the absence of any commercial or financial relationships that could be construed as a potential conflict of interest.

## Footnotes

^1^https://www.ema.europa.eu/en/medicines/human/EPAR/rebif

^2^https://www.accessdata.fda.gov/drugsatfda_docs/label/2016/204063s014lbl.pdf

^3^https://www.ema.europa.eu/en/documents/product-information/mayzent-epar-product-information_en.pdf

^4^https://www.ema.europa.eu/en/documents/product-information/tysabri-epar-product-information_it.pdf

## Abbreviations

AAR: annualized relapse rate
ALZ: alemtuzumab
MS: multiple sclerosis
BBB: blood–brain barrier
DMF: dimethyl fumarate
DMT: disease-modifying therapy
EMA: European Medicine Agency
FDA: Food and Drug Administration
GA: glatiramer acetate
IFN β: interferon beta
NAT: natalizumab
PPMS: primary progressive multiple sclerosis
OCR: ocrelizumab
RRMS: relapsing–remitting multiple sclerosis.
